# Interleukin-2 receptor β chain as a possible target for low doses of mafosfamide

**DOI:** 10.1155/S0962935195000287

**Published:** 1995-05

**Authors:** A. Pukhalsky, A. Toptygina, S. Khaidukov

**Affiliations:** 1 Research Centre for Medical Genetics Moskvorechie St. Moscow 115478 Russia; 2 M.M. Shemyakin and Yu.A. Ovchinnikov Institute of Bioorganic Chemistry Moscow Russia

## Abstract

The 7-day cytotoxic lymphocytes (CTL) induced in mixed lymphocyte culture express only the chain of the interleukin-2 receptor (IL-2R). In the present study this fact has been confirmed in a murine semi-allogeneic system. The ability of low doses of mafosfamide (Mf) to affect IL-2-induced CTL proliferation has been demonstrated. It was also shown that IL-2 activated resting suppressor cells. The pretreatment of the suppressor cells with either monoclonal antibodies (mAbs) against the p75 chain of IL-2R, or with Mf abolished the suppressive effect of these cells. No restoration of the proliferative response occurred when the anti-IL-2Rα mAb had been used. Flow cytometry analysis of 7-day CTL was carried out with mAbs against the α and β chains of IL-2R. CTL treatment with Mf inhibited anti-IL-2Rβ mAb binding. It may be assumed that the anti-proliferative effects of Mf which have been demonstrated in this paper, were a result of blocking the IL-2R β chain.


Mediators of Inflammation 4, 175–180 (1995)

### Interleukin-2 receptor β chain as a possible target for low doses of mafosfamide

A. Pukhalsky,[1,CA] A. Toptygina[1] and S. Khaidukov[2]

[1]Research Centre for Medical Genetics, 1 Moskvorechie St., 115478, Moscow; [2]M.M. Shemyakin and Yu.A. Ovchinnikov Institute of Bioorganic Chemistry, Moscow, Russia

[CA]Corresponding Author

**THE 7-day cytotoxic lymphocytes (CTL) induced in mixed lymphocyte culture express only the β chain of the interleukin-2 receptor (IL-2R). In the present study this fact has been confirmed in a murine semi-allogeneic system. The ability of low doses of mafosfamide (Mf) to affect IL-2-induced CTL proliferation has been demonstrated. It was also shown that IL-2 activated resting suppressor cells. The pretreatment of the suppressor cells with either monoclonal antibodies (mAbs) against the p75 chain of IL-2R, or with Mf abolished the suppressive effect of these cells. No restoration of the proliferative response occurred when the anti-IL-2Rα mAb had been used. Flow cytometry analysis of 7-day CTL was carried out with mAbs against the α and β chains of IL-2R. CTL treatment with Mf inhibited anti-IL-2Rβ mAb binding. It may be assumed that the anti-proliferative effects of Mf which have been demonstrated in this paper, were a result of blocking the IL-2R β chain.**

**Key words:** Alkylating agents, Cytotoxic lymphocytes, Interleukin-2 receptors, Mafosfamide, Suppressor cells

### Introduction

Interleukin-2 (IL-2) is a T-cell derived lymphokine which participates in the manifestation of the normal immune response. IL-2 exerts its biological activities by binding to a specific receptor on a target cell.[1] The IL-2 receptor (IL-2R) is composed of at least three cell–surface proteins, designated as the α-, β-, and γ-subunits. Different combinations of these subunits can define a number of states with differing affinities for IL-2 at the cell surface.[2–10]

The interaction of IL-2 with its multi-subunit receptor can be a target for drugs with immunomodulating activities. For adequate immunocorrection it is very important to know what kind of IL-2R chain is affected by the immunosuppressive drug. For example, it has been shown that lymphocytes treated *in vitro* with cyclosporin A (CsA) show significant inhibition of the p55 chain (α subunit) of IL-2R.[11–13]

Previously, our group has shown that the spleen cells from mice of various genotypes have a different susceptibility to the antiproliferative action of alkylating agents.[14] The different sensitivity of lymphoid cells from different strains of mice to mafosfamide (a synthetic analogue of the alkylating metabolites of cyclophosphamide), and to the antiproliferative effects of cyclosporin A, has been also demonstrated. These interstrain variations have not been related to the inhibition of interleukin 2 (IL-2) release but depend on differences in the expression of the IL-2 receptor (IL-2R). On the basis of these experiments we considered that the β chain (p75) of IL-2R was a possible target for mafosfamide (Mf) when administered in doses which were relatively low but sufficient for the inhibition of lymphocyte proliferation.

To test this hypothesis we needed an experimental system where the lymphoid cells expressed only the β subunit of IL-2R. Two experimental systems were used.

It is known that amongst cytotoxic lymphocytes (CTL) there exists a subset which expresses the β chain but not the α chain of IL-2R.[16] Our unpublished data show that within CTL induced in a mixed lymphocyte culture, both α and β chains of IL-2R occur, but beginning on the seventh day of cultivation the cells express the p75 chain only. This seventh day CTL culture was used as the first experimental system.

It is now evident that $CD8^+$ cells reveal a significantly greater number of binding sites for anti-p75 than for anti-p55 monoclonal antibodies (mAbs).[17] Cell populations of thymocytes and splenocytes with phenotype $CD3^+$, $CD4^-$, $CD8^+$ express IL-2Rβ.[18] These data support the idea that resting suppressor cells exist among normal spleen cells and express the β chain of IL-2R. This idea formed the basis of the second experimental system.

© 1995 Rapid Communications of Oxford Ltd

### Material and Methods

*Mice*: BALB/cJlac, DBA/2J, CC57BR/Mv, C57BL/6J, and (C57BL/6 × DBA/2)$F_1$ mice were obtained from the Russian Academy of Medical Sciences Care Units 'Stolbovaya' and 'Rappolovo' (CC57BR). Hybrids (BALB/c × CC57BR)$F_1$ were obtained from the vivarium of the Research Centre for Medical Genetics. Male mice weighing 22–24 g were used.

*Immunosuppressive agents*: Mafosfamide (Asta Z7654, Asta-Werke, Germany) and cyclosporin A (Sandimiun, Sandoz, Switzerland) were used as immunosuppressants.

*Cell cultures*: The isolation of lymphoid cells from murine spleen and the inhibition of Con A-induced lymphocyte proliferation by mafosfamide (Mf) and cyclosporin A (CsA) have been described in our previous publications.[14,19] Briefly, mice were killed by cervical dislocation. Lymphoid cells were isolated from the spleens, washed, and resuspended in RPMI-1640 medium (Flow Lab., UK) supplemented with 10% horse serum, 2 × $10^{-3}$ M HEPES, 2 mM L-glutamine, 2.8 × $10^{-6}$ M 2-mercaptoethanol, and 20 μg/ml gentamicin. Inhibition of Con-A stimulation by mafosfamide or cyclosporin A was evaluated at six different concentrations within the dose ranges 0.1 – 30 μg/ml and 0.03 – 10 μg/ml, respectively. The cells were incubated in flat-bottomed 96-well plates (Nunc, Denmark) with different concentrations of the drugs for 1 h at 37°C in humidified atmosphere containing 5% $CO_2$. Then the plates were centrifuged, the supernatants were washed away by Transtar-96 (Costar, USA) and fresh culture medium with Con-A was added. The control wells incubated without drugs contained a culture medium with Con-A or culture medium only. The cells were incubated for 72 h, pulsed with 40 kBq per well of [$^3$H]-thymidine 4 h before the end of cultivation, harvested with a cell harvester, and counted by using a liquid scintillation counter.

Cytotoxic lymphocytes (CTL) were induced in a semi-allogeneic mixed lymphocyte culture (Fig. 1(a)). When lymphocytes of C57BL/6 and DBA/2 mice were used as responding populations, (C57BL/6 × DBA/2)$F_1$ mice served as a source of stimulator cells. The lymphocytes of (BALB/c × CC57BR)$F_1$ were used as stimulator cells for the lymphocytes of BALB/c and CC57BR mice. Both the responder and stimulator cells ($10^6$ cells of each population) were co-cultivated for 7 days at 37°C in a humidified atmosphere containing 5% $CO_2$ in the wells (2 ml per well) of 24-well plates (Nunc, Denmark). The cells were washed twice and incubated for 1 h at a con-

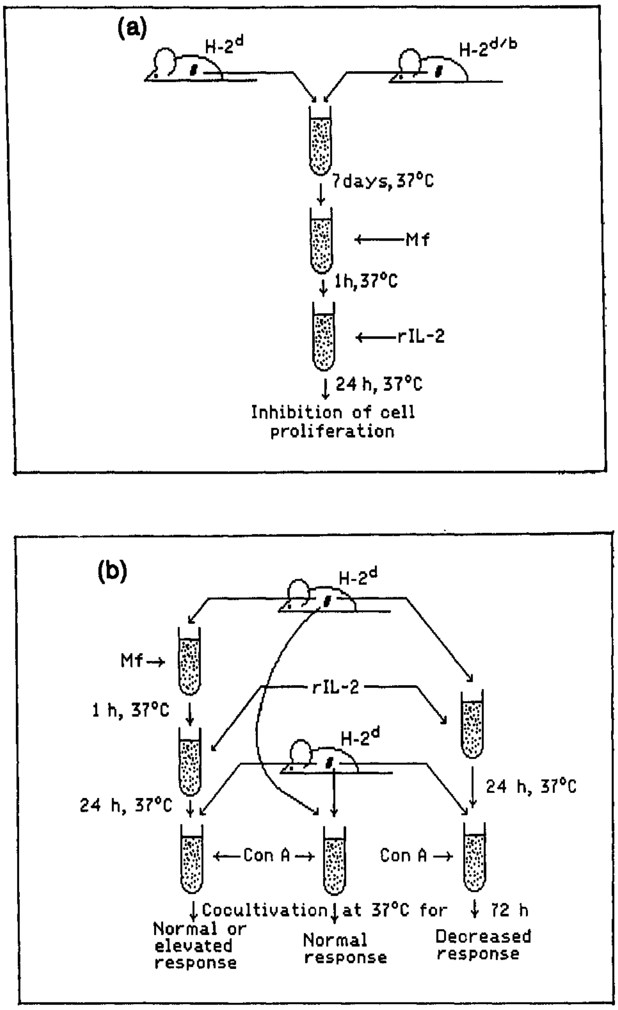

FIG. 1. Inhibition of CTL proliferative response (a) and suppressor cell activity (b) with Mf treatment. Schemes of experiments (see text).

centration of 2 × $10^5$ cells per well in flat-bottomed 96-well plates with or without different doses of Mf or CsA. Further, the cells were washed again and cultivated under the same conditions for 24 h in the presence of 10 IU/ml of recombinant IL-2 (rIL-2; Amersham, UK). Cell proliferation was evaluated by [$^3$H]-thymidine incorporation.

Suppressor cells were activated by recombinant IL-2 (rIL-2) treatment (Fig. 1(b)). Spleen cells were incubated in the presence of 10 IU of rIL-2 (Sigma, USA) for 24 h. For the evaluation of the suppressor activity, rIL-2-treated cells were co-cultivated with the fresh isolated syngeneic spleen cells in a ratio of 1:1. Co-cultivation of normal cells with the cells incubated without rIL-2 was used as a control. The cell mixture was stimulated with Con A and incubated for 72 h. As a result, the fresh isolated cells co-cultivated with the cells incubated without rIL-2 showed a normal proliferative response to Con A. In contrast, the mixture of normal spleen cells and cells preincubated with rIL-2 demonstrated a significantly decreased response. The suppressor cell sensitivity to Mf was evaluated by pretreating the cells with different doses of the drug for an hour. Then cells were washed and cultivated in the presence of rIL-2 as described above.

*Flow cytometry:* α and β chains of IL-2R were detected by an indirect immunofluorescence method. A whole population of murine splenocytes was used. IL-2Rα was detected by a mAb to the IL-2R α chain (p55) (Becton & Dickinson, USA). Monoclonal antibodies (Tm-β1), specific for the β chain of IL-2R (p75), were a generous gift from Dr T. Tanaka, Tokyo Metropolitan Institute of Medical Science.[10] For indirect immunofluorescent staining, $1.2 \times 10^6$ spleen cells were washed three times with PBS containing 2% FCS and were incubated for 30 min on ice with 100 μl of monoclonal antibody diluted according to the manufacturer's instructions. Cells were washed with PBS/FCS and treated under the same conditions with FITC-labelled $F(ab')_2$-fragments of rabbit anti-mouse Ig antibodies (DAKOPATTS, Denmark). After washing with PBS/FCS, cells were analysed by using flow cytometry. An EPICS 'ELITE' flow cytometer (Coulter, USA) was used. At least $10^5$ cells were analysed and the data processed by means of the MultiGraph program (Coulter, USA).

*Statistical analysis:* Statistical comparisons were performed using the Wilcoxon–Mann–Whitney's *U* criterion, paired Student's *t*-test, and probit-analysis for $ED_{50}$ calculations.

### Results

*The influence of Mf and CsA on CTL:* Mf strongly suppressed the response of 7-day CTL to IL-2. In contrast, CsA did not suppress IL-2 stimulated CTL; nevertheless, we have shown a strong inhibition of Con A-stimulated spleen cell proliferation at significantly lower doses. The inhibition of IL-2-stimulated CTL with Mf was carried out in the same dose range as the spleen cell proliferative response induced with Con A (Fig. 2). Thus, the cell sensitivities for DBA/2 and C57BL//6 mice were significantly higher than those for BALB/c and CC57BR ($p < 0.05$). It is obvious that this model reveals the same inter-strain differences as the experiments with the freshly isolated Con A-stimulated spleen cells.

*Blocking of the suppressor cell activity by mAbs and Mf:* The ability of IL-2 to stimulate suppressor cells was demonstrated. Thus, the significant decrease of the proliferative response level was a result of co-cultivation of freshly isolated Con A-stimulated murine spleen cells with syngeneic lymphocytes preincubated with IL-2 for 24 h (Figs. 3 and 4). The pretreatment of suppressor cells with anti-p75 mAb restored the normal lymphocyte response level. No restora-

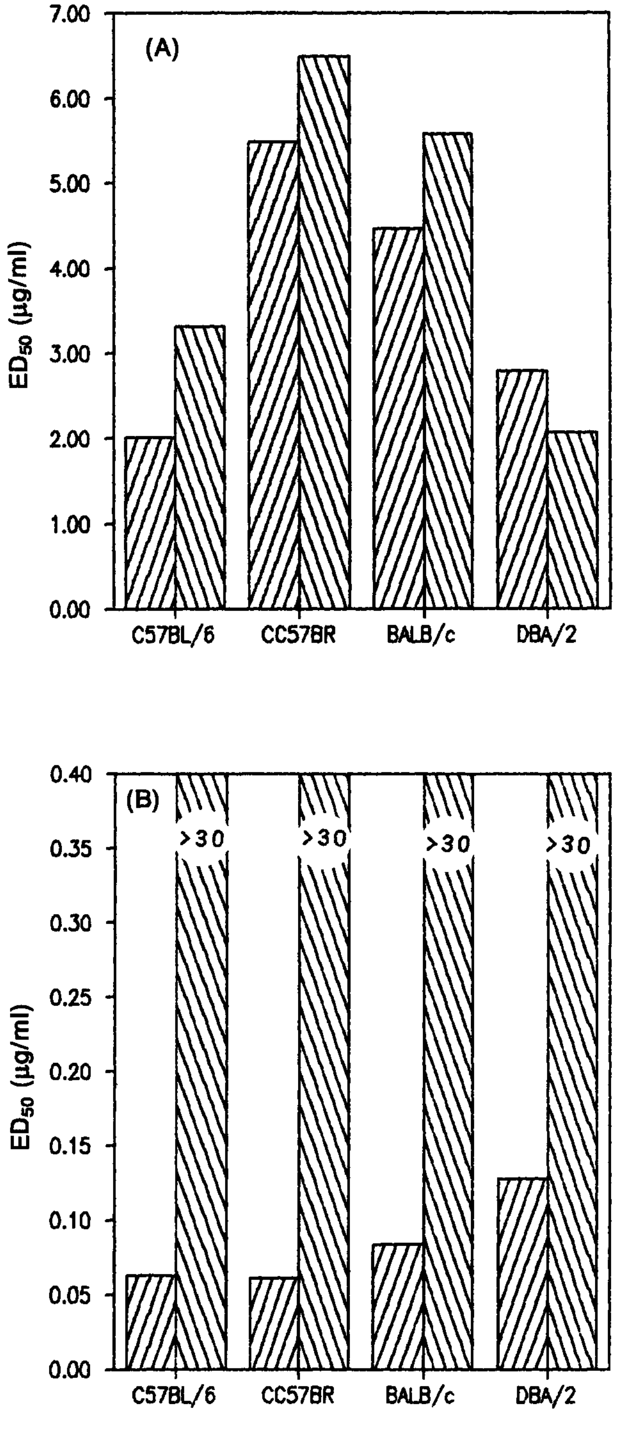

FIG. 2. Mf (A) and CsA (B) action on the proliferative response of Con A-stimulated freshly isolated spleen cells or IL-2-stimulated 7-day CTL culture. ▨, spleen cells; ▧, cytotoxic lymphocytes.

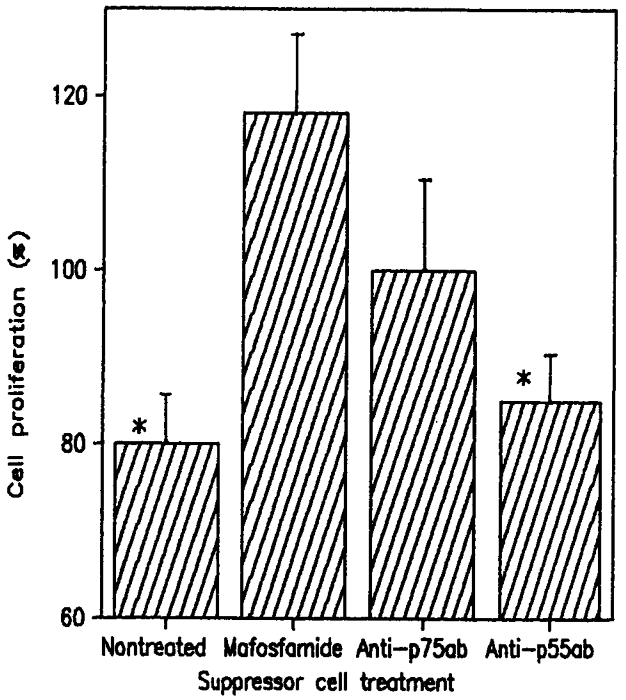

FIG. 3. Restoration of proliferative response to Con A decreased by IL-2-stimulated suppressor cells with MF or anti-p75 monoclonal antibodies in DBA/2 mice. Proliferative response of spleen cells cultivated without IL-2-stimulated cells is taken as 100%. *$p < 0.05$.

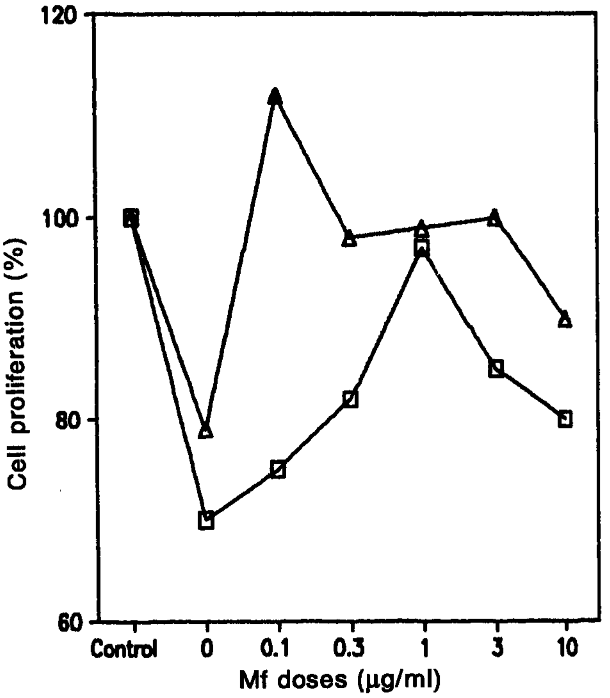

FIG. 4. Different sensitivity of suppressor cells to Mf in BALB/c (□) and C57BL/6 (△) mice (see text).

tion of the proliferative response was demonstrated with anti-p55 mAb. This level was also increased after the pretreatment of suppressor cells with low doses of Mf. The restoration of the proliferative response was dose-dependent and strain-specific. Our data demonstrated that exposure of C57BL/6 cells to Mf at 0.1 μg/ml resulted in the maximum increase of lymphocyte proliferation. In BALB/c mice, a dose which ensured a maximum level of restoration was ten times higher than in C57BL/6 mice (see Fig. 4).

*The influence of Mf on mAb binding with α and β chains of IL-2R:* The results obtained in this study are shown in Fig. 5 which demonstrates the binding of mAbs specific for α or β chains of IL-2R with 7-day CTL stimulated with rIL-2. The cells analysed by flow cytometry were stained with TM-β1 but not with the IL-2R α-chain-specific mAb. Thus, we observed about 7% of TM-$\beta1^+$ cells. It is necessary to bear in mind that the whole population of splenocytes contains about 30% T cells. This means that 25% T cells were TM-$\beta1^+$. Our data show that Mf has a dose-dependent effect on TM-β1 antibody binding (Fig. 5(a)). In addition, a nearly two-fold decrease of the TM-$\beta1^+$ cell level after the CTL incubation with rIL-2 for 24 h was demonstrated. These residual cells were also sensitive to Mf treatment (Fig. 5(b)).

### Discussion

Our results show that 7-day CTL express the β chain but not the α chain of IL-2R (see Fig. 5). Thus, in this study cells that expressed only constitutive IL-2R were investigated. It was shown that the exposure of CTL expressing the β chain of IL-2R to relatively low doses of Mf resulted in a strong inhibition of the proliferative response to IL-2, whereas these cells were insensitive to the action of CsA. The different sensitivity of CTL to Mf and CsA may be explained by the lack of a specific target for CsA on 7-day CTL. The appearance of the target (the p55 chain of IL-2R) on the fresh isolated spleen cells stimulated with Con-A makes these cells sensitive to the antiproliferative effect of CsA. The spleen cell response to ConA was inhibited with the same doses of Mf as was the CTL response to IL-2. The experiments showed that both these cell populations revealed the same interstrain variations (see Fig. 2).

Our data indicate the widely accepted opinion that the mechanism of action of alkylating agents is the result of DNA–DNA linkage,[20] but does not

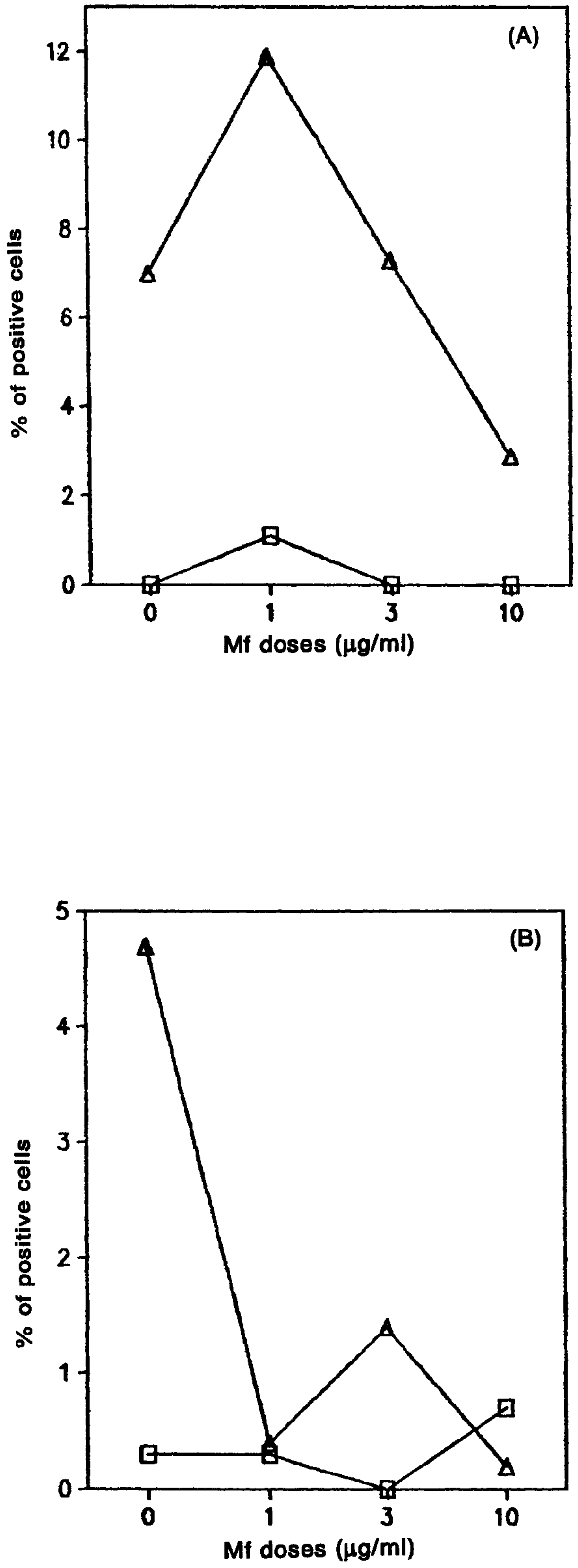

FIG. 5. Flow cytometry analysis of CTL staining with mAbs specific to IL-2Rα (anti-p55, (□)) and IL-2Rβ (anti-p75, (△)) before and after treatment with Mf. The 7-day CTL (A) and 7-day CTL incubated with rIL-2 during 24 h (B) were examined. The number of labelled cells is presented as a percent of total spleen cells counted.

explain completely the mechanisms of drug activity. It seems that the aforesaid mechanism is important for the suppression of malignant cell proliferation with high doses of alkylating drug, when there is no other alternative but irreversible damage to cell reproduction. In our case, the alkylating drug plays the role of immune response modifier but not the role of crucial cytostatic. The present data indicate that Mf can not only damage the proliferative response of cells but can also restore it by means of suppressor cell inhibition (see Figs. 3 and 4), it being shown that the suppressor cell activation was carried out by IL-2Rβ. These data help to explain the well-known fact that the suppressor cells are a most sensitive subset to cyclophosphamide.[21–23]

It should be noted that the increase in the proliferative response was more visible in C57BL/6 than in BALB/c mice (see Fig. 4). Previously, it was shown that the constitutive IL-2R were expressed in BALB/c mice clearly, whereas their expression in C57BL/6 mice was poor.[15] These data explain why Mf was able to affect cell suppressor activity in C57BL/6 to a higher degree than in BALB/c mice.

In conclusion, our data suggest that the antiproliferative effect of Mf on lymphocytes activated with alloantigens or mitogens is a result of blocking the IL-2R β chain. Thus, an additional mode of action of alkylating drugs has been shown.

### References

1. Waldmann TA. The IL-2/IL-2R receptor system: a target for rational immune intervention. *Immunol Today* 1993; **14**: 264–269.
2. Leonard WJ, Depper JM, Crabtree GR, *et al.* Molecular cloning and expression of cDNAs for the human interleukin-2 receptor. *Nature* 1984; **311**: 626–629.
3. Nikaido T, Shimizu A, Ishida N, *et al.* Molecular cloning of cDNA encoding human IL2 receptor. *Nature* 1984; **311**: 631–635.
4. Hatakeyama M, Tsudo M, Minamoito S, *et al.* Interleukin-2 receptor β-chain gene: generation of three receptor forms by cloned human α and β chain cDNAs. *Science* 1989; **244**: 551–556.
5. Hatakeyama M, Mori H, Doi T, Taniguchi T. A restricted cytoplasmic region of IL2 receptor β chain is essential for growth signal transduction but not for ligand binding and internalization. *Cell* 1989; **59**: 837–845.
6. Tsudo M, Karasuyama H, Kitamura F, *et al.* The interleukin-2 receptor β chain (p70): ligand binding ability of the cDNA encoding membrane and secreting forms. *J Immunol* 1990; **145**: 599–606.
7. Minamoto S, Mori H, Hatakayama M, *et al.* Characterisation of heterodimeric complex of human IL-2 receptor α–β chains reconstituted in a mouse fibroblast cell line. *J Immunol* 1990: **145**: 2177–2182.
8. Takeshita T, Asao H, Ohtani K, *et al.* Cloning of the γ chain of the human IL-2 receptor. *Science* 1992; **257**: 379–382.
9. Nakamura Y, Russell SM, Mess SA, *et al.* Heterodimerization of the IL-2 receptor β and γ chain cytoplasmic domains is required for signalling. *Nature* 1994; **369**: 330–333.
10. Tanaka T, Tsudo M, Karasuyama H, *et al.* A novel monoclonal antibody against murin IL-2 receptor β chain. Characterization of receptor expression in normal lymphoid cells and EL-U cells. *J Immunol* 1991; **147**: 2222–2228.
11. Reed JC, Abidi AH, Alpers JD, Hoover RG, Roob RJ, Novel PC. Effects of cyclosporin A and dexamethazone in interleukin 2 receptor gene expression. *J Immunol* 1986; **137**: 1540–154.
12. Bloemena E, Van Ders MHJ, Weinreich S, Schellekens P. Cyclosporin A and prednisolone do not inhibit the expression of high-affinity receptor for interleukin-2. *Clin Exp Immunol* 1988; **71**: 308–313.

13. Caillat-Zucman S, Chatenoud L, Bach J-F. *In vitro* and *in vivo* action of cyclosporin A on the induction of human interleukin-2 receptor α and β chains. *Clin Exp Immunol* 1989; **77**: 184–190.
14. Pukhalsly AL, Toptygina AP, Viktorov VV. Immunosuppressive action of cyclophosphamide in mice: contribution of some factors to determination of strain differences. *Int J Immunopharmac* 1993; **15**: 509–514.
15. Pukhalsky AL, Toptygina AP. The comparative study of mechanisms of antiproliferative effect of mafosfamide and cyclosporin A applied in low doses. *Bull Exp Biol Med* 1993; **115**: 514–515.
16. Kiziroglu F, Miller RG. Effect of cyclosporine on secondary cytotoxic T lymphocyte responses. *Transplantation* 1990; **49**: 961–965.
17. Ohashi Y, Takashita T, Nagata K, Mori S, Sugamura K. Differential expression of the IL-2 receptor subunits, p55 and p75 on various populations of primary peripheral blood mononuclear cells. *J Immunol* 1989; **143**: 3548–3555.
18. Hattori M, Okazaki H, Ishida Y, *et al.* Expression of murin IL-2 receptor β chain on thymic and splenic lymphocyte subpopulations as revealed by the IL-2 induced proliferative response in human IL-2 receptor α-chain transgenic mice. *J Immunol* 1990; **144**: 3809–3815.
19. Pukhalsky AL, Mezhneva AP, Pevnitsky IA. Sensitivity of splenocytes of mice of different strains to the antiproliferative effect of alkylating drugs. *Bull Exp Biol Med* 1988; **105**: 196–198.
20. Surya YA, Rosenfeld, JM, Hillcoat BL. Cross-linking of DNA in L-1210 cells and nuclei treated with cyclophosphamide and phosphoramide mustard. *Cancer Treat Rep* 1978; **62**: 23–29.
21. Duclos H, Galanaud P, Devinsky O, *et al.* Enhancing effect of low dose cyclophosphamide treatment on the *in vitro* antibody response. *Eur J Immunol* 1977; **7**: 673–684.
22. Diamanstein T, Willinger E, Reiman J. T-suppressor cells sensitive to cyclophosphamide and its *in vitro* active derivative 4-hydroperoxycyclophosphamide control the mitogenic response of murine splenic B cells to dextran sulphate. A direct proof for different sensitivities of lymphocyte subset to cyclophosphamide. *J Exp Med* 1979; **150**: 1571–1576.
23. Hoover SK, Barett SK, Turk TMT, Lee T-C, Bear HD. Cyclophosphamide and abrogation of tumor-induced suppressor T cell activity. *Cancer Immunol Immunother* 1990; **31**: 121–127.

ACKNOWLEDGEMENTS. This work was supported in part by a grant from the Russian State Program 'Frontiers in Genetics'. The authors are grateful to Dr T. Tanaka for providing antibodies against the β chain of IL-2R.

**Received 19 December 1994; accepted in revised form 3 February 1995**
